# Transcriptomic Profiling of Dromedary Camels Immunised with a MERS Vaccine Candidate

**DOI:** 10.3390/vetsci8080156

**Published:** 2021-08-03

**Authors:** Sharif Hala, Paolo Ribeca, Haya A. Aljami, Suliman A. Alsagaby, Ibrahim Qasim, Sarah C. Gilbert, Naif Khalaf Alharbi

**Affiliations:** 1 Vaccine Development Unit, Department of Infectious Disease Research, King Abdullah International Medical Research Center (KAIMRC), Riyadh 11481, Saudi Arabia; halash@ngha.med.sa (S.H.); aljamiha@ngha.med.sa (H.A.A.); 2 King Saud bin Abdulaziz University for Health Sciences, Riyadh 11481, Saudi Arabia; 3 Pathogen Genomics Laboratory, King Abdullah University of Science and Technology (KAUST), Thuwal 23955, Saudi Arabia; 4 Biomathematics and Statistics Scotland, The James Hutton Institute, Edinburgh EH9 3FD, UK; paolo.ribeca@bioss.ac.uk; 5 Department of Medical Laboratory Sciences, College of Applied Medical Sciences, Majmaah University, Al Majmaah 11952, Saudi Arabia; s.alsaqaby@mu.edu.sa; 6 Ministry of Environment, Water and Agriculture (MEWA), Riyadh 11481, Saudi Arabia; i.qasim@mewa.gov.sa; 7 The Jenner Institute, Nuffield Department of Medicine, University of Oxford, Oxford OX1 4BH, UK; sarah.gilbert@ndm.ox.ac.uk

**Keywords:** transcriptome, gene expression, camel, MERS-CoV, vaccine, immunogenicity

## Abstract

Middle East Respiratory Syndrome coronavirus (MERS-CoV) infects dromedary camels and zoonotically infects humans, causing a respiratory disease with severe pneumonia and death. With no approved antiviral or vaccine interventions for MERS, vaccines are being developed for camels to prevent virus transmission into humans. We have previously developed a chimpanzee adenoviral vector-based vaccine for MERS-CoV (ChAdOx1 MERS) and reported its strong humoral immunogenicity in dromedary camels. Here, we looked back at total RNA isolated from whole blood of three immunised dromedaries pre and post-vaccination during the first day; and performed RNA sequencing and bioinformatic analysis in order to shed light on the molecular immune responses following a ChAdOx1 MERS vaccination. Our finding shows that a number of transcripts were differentially regulated as an effect of the vaccination, including genes that are involved in innate and adaptive immunity, such as type I and II interferon responses. The camel Bcl-3 and Bcl-6 transcripts were significantly upregulated, indicating a strong activation of Tfh cell, B cell, and NF-κB pathways. In conclusion, this study gives an overall view of the first changes in the immune transcriptome of dromedaries after vaccination; it supports the potency of ChAdOx1 MERS as a potential camel vaccine to block transmission and prevent new human cases and outbreaks.

## 1. Introduction

Middle East Respiratory Syndrome coronavirus (MERS-CoV) causes a respiratory disease in humans that can vary from mild to severe pneumonia and organ failure with a ~35% mortality rate [1]. The virus circulates in dromedary camels in the Arabian peninsula, some parts of middle Asia, and Africa, causing zoonotic spillover cases in humans [2,3]. MERS-CoV has infected more than 2450 individuals in 27 countries with outbreaks that occurred mainly in the Arabian Peninsula in large crowded hospitals and one large outbreak in the Republic of Korea [1]. No other cases or outbreaks occurred in Korea, likely because of a small, to no, dromedary population [4]. This emphasises the importance of improving public health measures, especially in areas where camels are kept, such as camel markets and festivals, and the need for a MERS vaccine for camels [5]. More than half (54.9%) of the primary human cases, which were mostly in Saudi Arabia, have reported contact with camels [6]; and the index patient in the Korean outbreak traveled back from the Arab Gulf countries where MERS-CoV is endemic and circulating in dromedaries before causing the outbreak [2,7,8].

There have been international efforts to develop therapeutics and vaccines, supported by putting the need for a MERS vaccine on the priority lists of the WHO and CEPI [9,10]. To date, however, there is no approved antiviral or vaccine for MERS-CoV in humans or camels. In camels, the MERS-CoV infection is mild, transient, and does not require veterinary care. However, a scientific proposition was put forward that developing a vaccine for camels would prevent, or at least reduce, transmission into humans, predominantly asymptomatic and mild human cases found among people in contact with camels [5,6,9]. Preventing the infection in this population would block the virus transmission into the community, especially patients with co-morbidities who are more likely to develop severe MERS cases. Therefore, successful vaccination of camels would reduce virus circulation in this animal host, reducing human exposure, while the same vaccine may be developed for humans to control the endemic further; this is often called a one-health approach [11].

Several vaccine candidates have been developed and tested in multiple animal models, including mice, rabbits, non-human primates, and dromedaries [9,10,12]. While many of these vaccines were developed for humans, three have been tested in dromedaries as one-health vaccines [13,14,15]. We have previously reported the development of a chimpanzee adenoviral vector-based vaccine for MERS-CoV (ChAdOx1 MERS) that has now been tested in mouse models for immunogenicity [16] and efficacy [17]; it was further evaluated for safety and immunogenicity in humans [18].

The ChAdOx1 MERS vaccine was trialed in dromedaries, where it induced robust antibody responses and showed a significant partial protection [13]. However, there were some technological difficulties when assessing T cell responses in camels, mainly for the lack of T cell markers for flow cytometric analysis and ELISpot assays. Immune responses could be gauged by the level of immune-related gene expression, increasing post-vaccination, which could explain immune responses elicited by the vaccine. Here, in order to examine dromedary immune responses at the transcriptomic level, three camels that were immunised with ChAdOx1 MERS were sampled pre-vaccination and at three time points post-vaccination. Total RNA was isolated from whole blood and sequenced to report the differentially expressed genes and their implications for the camel immune system. Our aims were to observe the transcriptomic immune signature changes in order to provide better insights for future exploration of markers linked with protection.

## 2. Results

### 2.1. The Study Animals

To observe changes in the immune system of three dromedary camels that were free of MERS infection prior to being immunised with ChAdOx1 MERS vaccine candidates, we investigated the animals’ transcriptomic profile of whole blood at four time points; 0 h (pre-vaccination) 6, 12, and 24 h post-vaccination. The calves were ensured to be MERS-CoV infection-free by negative PCR results. They were not exposed to MERS-CoV before, as confirmed by negative ELISA testing and negative neutralisation assays mentioned in previous work [13].

### 2.2. Changes in Overall Gene Expression

**Figure 1:**
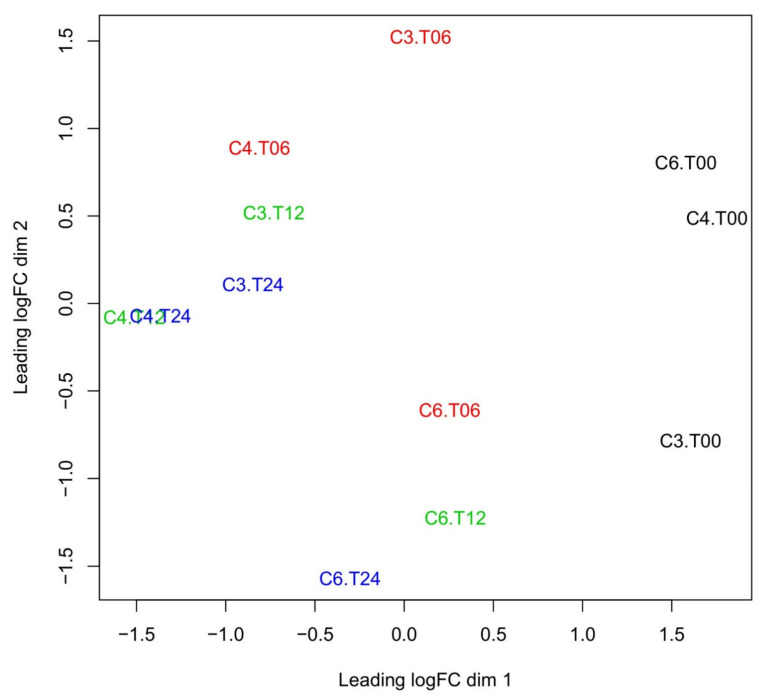
**Multidimensional scaling (MDS) analysis of transcription log-fold changes.** Each label corresponds to a camel (C) and a time point (T). There are three camels (C3, C4, and C6) and four times (T) points: 0, 6, 12, and 14 h post-vaccination). MDS analysis shows a detectable change in the overall gene regulation after vaccination. Change in time occurs mostly along the leading MDS dimension 1 (x-axis). An individual effect is observed (mostly along the second MDS dimension 2, y-axis). Regulation at each time point is similar for all camels, leading to points for the same time (black = 0 h, red = 6 h, green = 12 h, blue = 24 h after vaccination) being relatively clustered together. However, some exceptions do exist, notably for camel 4.

Before and after the vaccination of camels, the overall gene expression at a global transcriptomic level of whole blood was assessed using multidimensional scaling (MDS) for all camels and time points (Figure 1). The MDS plot confirmed a general time-dependent expression pattern in all animals (C3, C4, and C6), with the expressions at a given time broadly clustering together both before and after vaccination. However, some distinct differences between individual camels are also present, especially at later time points, possibly indicating gene regulation effects due to age and breed; age has been recently shown to impact the immune-related transcriptome in humans [19].

### 2.3. Time-Dependent Behavior of Gene Regulation

The general transcriptional fingerprint had a total number of 14,290 transcripts. Considering the differential regulation with more than 1 log2 of fold-change, there were: 1005 upregulated and 392 downregulated transcripts at 6 h post-vaccination; 1064 upregulated and 352 downregulated transcripts at 12 h post-vaccination, and 791 upregulated and 248 downregulated transcripts at 24 h post-vaccination (Supplementary Files 2–4). However, the change in transcript abundance was not always statistically significant.

**Figure 2:**
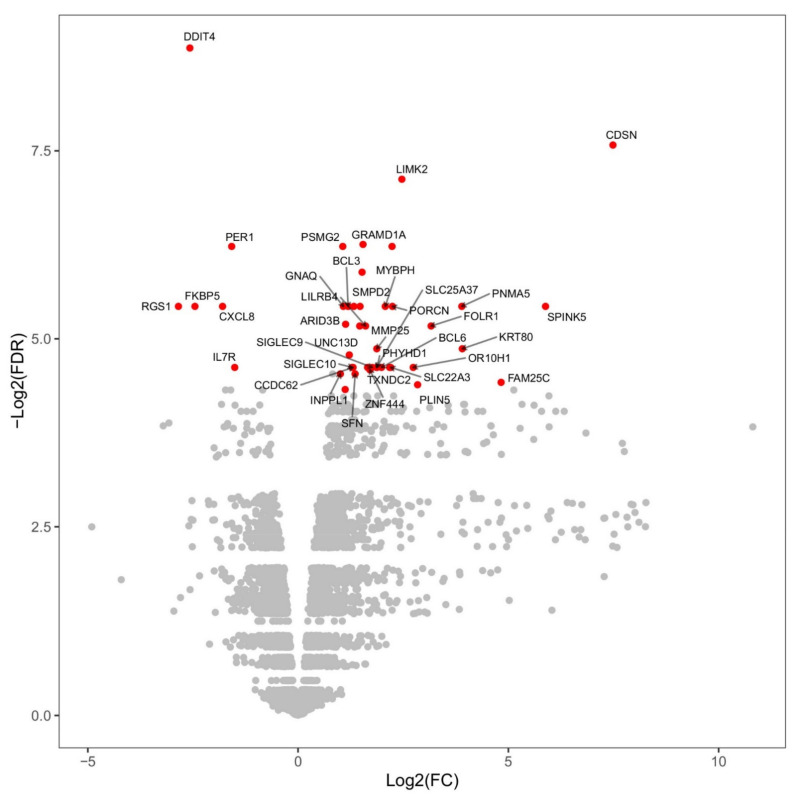
**Volcano plot for gene regulation at 6 h post-vaccination**. Changes measured at 6 h post-vaccination as compared to time 0 h (i.e., pre-vaccination). On the x-axis, the base-2 logarithm of the fold-change; on the y axis, the negative base-2 logarithm of the false discovery rate (FDR) for the fold-change. The dots in red are those that exhibit statistically significant differential regulation at 6 h, defined as abs (log2(fold change)) ≥ 1 and FDR ≤ 0.05. The long versions of the gene annotations corresponding to the short gene names appearing in the plot can be found in Table S1.

A comparison between gene expression before vaccination and gene expression at 6 h post-vaccination can be found in the volcano plot of Figure 2, along with the statistical significance of the change for each gene. A total of 40 transcripts had statistically significant changes, of which 6 transcripts were downregulated, including CXCL8 and IL7R, and 34 transcripts were upregulated, including Bcl-6 and Bcl-3 (≥2-fold change, and False Discovery Rate  ≤  0.05). Bcl-6 and Bcl-3 are transcription factors involved in the activation of B cells and NF-κB, respectively. Therefore, in support of the previously reported immunogenicity study [13], this analysis indicates that a single dose of ChAdOx2-MERS elicits strong B and T cell-based immune responses.

**Figure 3:**
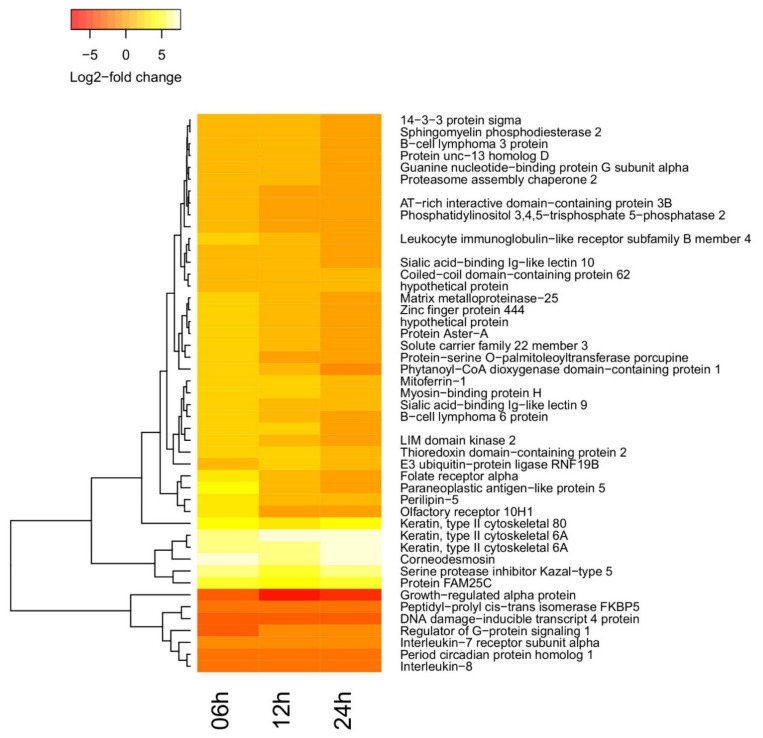
**Heatmap for the annotated camel genes.** Statistically significant differential regulation of genes at one or more time points post-vaccination. The full list of changes with corresponding statistical estimators can be found in Table S1.

A few more genes turn out to be differentially regulated (DR) in a statistically significant way at 12 and 24 h post-vaccination. The 47 transcripts exhibiting statistically significant changes in at least one condition compared to their pre-vaccination level are presented in the heatmap (Figure 3) and Supplementary File 1. Of those, four (Cadr_000005463, Cadr_000011353, Cadr_000023835, and Cadr_000025074) are putative transcripts whose function is not known, and two (Cadr_000007726 and Cadr_000002580) correspond to hypothetical proteins. For all other transcripts, it was possible to find mammalian orthologues with known biological functions.

### 2.4. Functional Enrichment of Differentially Regulated Genes

In general, upregulated transcripts associated with the immune response included genes related to the activation or expression of IL-1beta, IL-2, IL-6, IL-7, IL-10, IL-13, IL-17, IL-18, type I interferon, and interferon-gamma, indicating a strong innate immune response as well as activation of T cell immune responses via multiple pathways (Supplementary Files 2–4). The comparison of expression profiles between the different time points revealed immune-related genes both down- and upregulated, although not necessarily in a statistically significant way; more transcripts involved in the innate immune response were upregulated overtime. More interferon-related genes became highly upregulated by 24-h post-vaccination (Supplementary Files 2–4). Enrichment analysis of the 47 differentially regulated and statistically significant genes as a function of time are presented in Table 1. At all times, and especially 6- and 12-h post-vaccination, the main terms related to both innate and adaptive immune response. At 24 h post-vaccination, skin repair pathways also appear to have been activated; this is likely due to repair of the skin that is damaged by injection puncture as has been suggested previously [20].

## 3. Discussion

In this study, we reported the early changes in the transcriptome of dromedary camels following a vaccination experiment with a single dose of intramuscular ChAdOx1 MERS vaccine candidate. A previous study reported that this vaccine induced strong antibody responses, starting from day 7 post-vaccination and plateauing for over a month [13]. Here, the bioinformatic analysis of camel transcriptome during the first day after vaccination showed at least 47 DR genes with statistical significance. These genes are mainly involved in immune responses and skin damage and repair, indicating reactions towards the injection site. For immune-related genes, LILRB4, Bcl-3, Bcl-6, IL7R, CXCL1, and CXCL8 (IL8) were among the most DR genes. However, several other immune-related genes were upregulated over time following immunization; interferon-related genes, including type I and II (gamma) interferon.

Notably, CXCL1 and CXCL8, which is IL-8, were downregulated after vaccination in the camels being studied; our analysis indicates a marked decrease in the activation of both CXCL1 and CXCL8 from 6 h post-vaccination onward. As for several other chemokines, the expression of IL-8 in humans is associated with the recruitment of neutrophils and macrophages to infection or tumor sites, especially into tumor microenvironments [21,22,23,24,25]. In Influenza human vaccination studies, IL-8 levels decreased following the vaccine for at least 24 h [23] and this was more evident in non-pregnant women [21]; this is similar to our study in camels and warrant further investigation. On the other hand, human tumor studies showed that chemokines such as CXCL1 and CXCL8 are produced by tumor cells, macrophages, and neutrophils to recruit further neutrophils, macrophages, and sometimes myeloid-derived suppressor cells (MDSC) where the MDSC would prevent the killing of tumor cells [26]. However, the biology of immune stimulation of tumor microenvironments is complex, and it is unclear whether the early effects of camel vaccination would be similar to what happens in human tumors. In addition, these chemokines might have slightly different functions in the camel immune system. Supposing that function can be extrapolated from human to camel, our analysis hints at reduced neutrophil recruitment to the injection site in these early time points.

At such early time points post-vaccination, it might be less expected to detect gene regulation changes involved in adaptive immunity. However, our analysis showed an upregulation of Bcl-3 and Bcl-6; the latter is a transcription repressor that controls and regulates the activation and differentiation of Follicular helper T (Tfh) cells as well as modulates the activation of B cells by its involvement in the STAT-dependent IL-4 responses [27,28]. Although Bcl-6 is mainly involved in activation of germinal center (GC) Tfh cells and GC B cells, its expression in peripheral blood cells can be from macrophages or peripheral Tfc cells; this peripheral expression has been shown to contribute to Treg cell activation, induction of memory responses, and Tfh cells help to B cells outside the GCs [29,30,31,32]. Bcl-3, on the other hand, is a coactivator for NF-κB, which is a key player in the induction of immune responses to infections in humans, and it is a well-conserved gene in most vertebrates. Once activated, NF-κB leads to the activation and response of innate immunity as well as adaptive B and T cell responses [33]. Other significant DR genes in this study included leukocyte immunoglobulin-like receptor subfamily B member 4 (LILRB4), which was upregulated in the immunised camels; LILRB4 has a bidirectional activity in the activation of many immune cells and is involved in a complex immune process. It can elicit T cell activation, especially the regulatory T (Treg) cells or T suppressor cells, and can promote the maturation of dendritic cells [34,35].

Considering the utility and applications of the present data, studies on system vaccinology usually attempt to identify a transcriptomic signature that can help in predicting vaccine induced immunity or protection. One study, on humans, revealed that some modules of blood transcriptome, not at a single-transcript level, can correlates with vaccine induced antibodies. However, this remains vaccine dependent and differs across transcription modules, and a universal immune signature for vaccine induced immunity has not yet been found [36]. Recently, COVID-19 patients who previously had received a COVID-19 vaccine induced higher upregulation of genes, where more than 70% of upregulated transcripts were immune-related; however, the immune transcriptome was not enough to predict the mortality or protection of these subjects [19]. Although our analysis did not reveal significant changes in other immune signature genes in the first 24 h after vaccination, the generalised upregulation of a number of immune-related genes over time supports a picture presenting an apparent activation of innate and adaptive immunity to the ChAdOx1-MERS vaccination in camels. Previous studies have reported strong activation of both arms of the immune system following chimpanzee adenoviral vectored vaccines in humans [37]. It is essential to mention that our study did not include a sham vaccine control group and compared the changes to pre-vaccinated camels. Nonetheless, our study is one of the first ones assessing camel transcriptome changes after vaccination, and the presented data indicate the potency of ChAdOx1 MERS in activating immune responses in dromedaries at the molecular level. Remarkably, such changes are detectable even a few hours after vaccination. Such profiling sets out a baseline for evaluating the immunogenicity of ChAdOx1 vectored vaccines in camels by transcriptomics.

## 4. Materials and Methods

### 4.1. Experimental Design and Immunizations Vaccination Study

Three dromedary calves, aged between 1 and 2 years old, were immunised with the vaccine candidate, ChAdOx1 MERS, intramuscularly at a dose of 1 × 10^9^ infectious units, as described previously. The calves were given codes of C3, C4, and C6. Blood (3 mL) was collected into Tempus^TM^ Blood RNA Collection Tubes (Applied Biosystems, Waltham, MA, USA) at one-hour pre-vaccination and 6, 12, and 24 h post-vaccination. According to the manufacturer’s instructions, total RNA was isolated using Tempus™ Spin RNA Isolation kits (Applied Biosystems, Waltham, MA, USA). Total RNA was frozen at −80 °C until the time of the analysis.

### 4.2. RNA Sequencing

According to the manufacturer’s instructions, the Libraries were prepared using TruSeq stranded RNA prep kit version 2 (Illumina, San Diego, CA, USA), using 1.0 μg of total RNA input per sample with dual indexing. Samples were sequenced in a single pool using HiSeq4000 (Illumina, San Diego, CA, USA) to produce 150 bp paired-end reads. Reads were filtered with TrimGalore prior to running downstream analysis to remove low base call quality. Sequencing data has been deposited into the NCBI SRA under BioProject accession number PRJNA701734. The number of reads for each sample is listed in Table 2.

### 4.3. Bioinformatics Analysis

After quality control, the RNA-sequencing data were aligned to the CamDro3 assembly of the *C. dromedarius* genome (accession number GCA_000803125.3). Briefly, reads were processed with a data analysis workflow based on the GEM mapper [38], an updated version of the pipeline used to process the GEUVADIS consortium data [39]. The workflow allows for spliced mapping to the reference genome. It includes a sensitive de-novo intron discovery stage, thus alleviating the need for a high-quality annotation of the reference genome that is seldom available for non-model species. The percentage of paired alignments for each sample is reported in Table 2 (for all samples except camel 3 at time 0, most of the reads resulted in high-quality mappings to the reference). Subsequently, read counts for all the transcripts present in the annotation were obtained from the samples’ alignments.

### 4.4. Statistical Analysis

Differential regulation was evaluated with edgeR [40]. The MDS analysis (Figure 1) was also produced with edgeR. The enrichment analysis of Table 1 was generated with ClusterProfiler [41].

## Figures and Tables

**Table 1:** Gene ontology terms significantly enriched for the 47 differentially regulated genes identified by this study.

Gene Set	Timepoint	Enriched Ontologies
**BCL3, BCL6, UNC13D**	6 h	Germinal center formation
**CXCL8, CXCL1**	12 h	Chemokine activity, Chemokine receptor binding, Antimicrobial humoral immune response, Response to chemokine, Neutrophil migration, Granulocyte migration, Myeloid leukocyte migration, Cytokine activity, Leukocyte chemotaxis, Cellular response to biotic stimulus, G-protein coupled receptor binding, Cytokine receptor binding, Cell chemotaxis, Response to a molecule of bacterial origin
**DDIT4, PER1**	12 h, 24 h	Response to a steroid hormone, Negative regulation of phosphorylation
**CXCL8, KRT64**	24 h	Entry into the host, Interaction with host, Antimicrobial humoral immune response, Regulation of symbiosis
**CDSN, KRT64**	24 h	Keratinization, Cornification, Keratinocyte differentiation, Epidermal cell differentiation, Skin development, Epidermis development

**Table 2:** Read-related metrics for the sequencing samples used in this study. Both the number of reads and the number of high-quality mappings refer to paired-end reads.

Camel	0 h		6 h		12 h		24 h	
	Total	Mapping	Total	Mapping	Total	Mapping	Total	Mapping
3	23.5 M	73.4%	23.0 M	96.4%	23.0 M	94.4%	18.7 M	96.2%
4	18.1 M	93.3%	14.8 M	96.2%	27.1 M	94.2%	27.3 M	96.0%
6	25.5 M	94.5%	21.5 M	96.0%	31.8 M	95.3%	19.2 M	96.0%

## Data Availability

Sequencing data has been deposited into the NCBI SRA under BioProject accession number PRJNA701734.

## Supplementary Materials

The following are available online at https://www.mdpi.com/article/10.3390/vetsci8080156/s1, Supplementary file 1, 2, 3, and 4.

## References

[1] WHO (2020). WHO|Middle East Respiratory Syndrome Coronavirus (MERS-CoV).

[2] Alagaili A.N., Briese T., Mishra N., Kapoor V., Sameroff S.C., de Wit E., Munster V.J., Hensley L.E., Zalmout I.S., Kapoor A. (2014). Middle East respiratory syndrome coronavirus infection in dromedary camels in Saudi Arabia. mBio.

[3] Mackay I.M., Arden K.E. (2015). Middle East respiratory syndrome: An emerging coronavirus infection tracked by the crowd. Virus Res..

[4] (2015). Korea Centers for Disease Control and Prevention. Middle East Respiratory Syndrome Coronavirus Outbreak in the Republic of Korea, 2015. Osong Public Health Res. Perspect..

[5] Alshukairi A.N., Zheng J., Zhao J., Nehdi A., Baharoon S.A., Layqah L., Bokhari A., Al Johani S.M., Samman N., Boudjelal M. (2018). High Prevalence of MERS-CoV Infection in Camel Workers in Saudi Arabia. mBio.

[6] Conzade R., Grant R., Malik M.R., Elkholy A., Elhakim M., Samhouri D., Ben Embarek P.K., Van Kerkhove M.D. (2018). Reported direct and indirect contact with dromedary camels among laboratory-confirmed MERS-CoV cases. Viruses.

[7] Alharbi N.K., Ibrahim O.H., Alhafufi A., Kasem S., Aldowerij A., Albrahim R., Abu Ubaidah A., Resolution A., Bayoumi F.A., Al-Mansour A.M. (2020). Challenge infection model for MERS-CoV based on naturally infected camels. Virol. J..

[8] Kasem S., Qasim I., Al-Hufofi A., Hashim O., Alkarar A., Abu-Obeida A., Gaffer A., Al-Fadil A., Zaki A., Al-Rumaihi A. (2018). Cross-sectional study of MERS-CoV-specific RNA and antibodies in animals that have had contact with MERS patients in Saudi Arabia. J. Infect. Public Health.

[9] Alharbi N.K. (2016). Vaccines against Middle East respiratory syndrome coronavirus for humans and camels. Rev. Med. Virol..

[10] Yong C.Y., Ong H.K., Yeap S.K., Ho K.L., Tan W.S. (2019). Recent Advances in the Vaccine Development against Middle East Respiratory Syndrome-Coronavirus. Front. Microbiol..

[11] Margalida A., Bogliani G., Bowden C.G.R., Donázar J.A., Genero F., Gilbert M., Karesh W.B., Kock R.A., Lubroth J., Manteca X. (2014). Science and regulation. One Health approach to use of veterinary pharmaceuticals. Science.

[12] Zhou Y., Jiang S., Du L. (2018). Prospects for a MERS-CoV spike vaccine. Expert Rev. Vaccines.

[13] Alharbi N.K., Qasim I., Almasoud A., Aljami H.A., Alenazi M.W., Alhafufi A., Aldibasi O.S., Hashem A.M., Kasem S., Albrahim R. (2019). Humoral Immunogenicity and Efficacy of a Single Dose of ChAdOx1 MERS Vaccine Candidate in Dromedary Camels. Sci. Rep..

[14] Haagmans B.L., van den Brand J.M.A., Victor S.R., Volz A., Wohlsein P., Smits S.L., Schipper D., Bestebroer T.M., Okba N.M.A., Fux R. (2016). An orthopoxvirus-based vaccine reducesvirus excretion after MERS-CoV infectionin dromedary camels. Science.

[15] Muthumani K., Falzarano D., Reuschel E.L., Tingey C., Flingai S., Villarreal D.O., Wise M.C., Patel A., Izmirly A., Aljuaid A. (2015). A synthetic consensus anti-spike protein DNA vaccine induces protective immunity against Middle East respiratory syndrome coronavirus in nonhuman primates. Sci. Transl. Med..

[16] Alharbi N.K., Padron-Regalado E., Thompson C.P., Kupke A., Wells D., Sloan M.A., Grehan K., Temperton N., Lambe T., Warimwe G. (2017). ChAdOx1 and MVA based vaccine candidates against MERS-CoV elicit neutralising antibodies and cellular immune responses in mice. Vaccine.

[17] Munster V.J., Wells D., Lambe T., Wright D., Fischer R.J., Bushmaker T., Saturday G., van Doremalen N., Gilbert S.C., de Wit E. (2017). Protective efficacy of a novel simian adenovirus vaccine against lethal MERS-CoV challenge in a transgenic human DPP4 mouse model. NPJ Vaccines.

[18] Folegatti P.M., Bittaye M., Flaxman A., Lopez F.R., Bellamy D., Kupke A., Mair C., Makinson R., Sheridan J., Rohde C. (2020). Safety and immunogenicity of a candidate Middle East respiratory syndrome coronavirus viral-vectored vaccine: A dose-escalation, open-label, non-randomised, uncontrolled, phase 1 trial. Lancet Infect. Dis..

[19] Knabl L., Lee H.K., Wieser M., Mur A., Zabernigg A., Knabl L., Rauch S., Bock M., Schumacher J., Kaiser N. (2021). Impact of BNT162b first vaccination on the immune transcriptome of elderly patients infected with the B.1.351 SARS-CoV-2 variant. medRxiv.

[20] Maruyama S.R., Carvalho B., González-Porta M., Rung J., Brazma A., Gustavo Gardinassi L., Ferreira B.R., Banin T.M., Veríssimo C.J., Katiki L. (2019). Blood transcriptome profile induced by an efficacious vaccine formulated with salivary antigens from cattle ticks. NPJ Vaccines.

[21] Christian L.M., Porter K., Karlsson E., Schultz-Cherry S., Iams J.D. (2013). Serum proinflammatory cytokine responses to influenza virus vaccine among women during pregnancy versus non-pregnancy. Am. J. Reprod. Immunol..

[22] Leonard E.J., Yoshimura T. (1990). Neutrophil attractant/activation protein-1 (NAP-1 [interleukin-8]). Am. J. Respir. Cell Mol. Biol..

[23] Talaat K.R., Halsey N.A., Cox A.B., Coles C.L., Durbin A.P., Ramakrishnan A., Bream J.H. (2018). Rapid changes in serum cytokines and chemokines in response to inactivated influenza vaccination. Influenza Other Respir. Viruses.

[24] Van Damme J., Rampart M., Conings R., Decock B., Van Osselaer N., Willems J., Billiau A. (1990). The neutrophil-activating proteins interleukin 8 and beta-thromboglobulin: In vitro and in vivo comparison of NH2-terminally processed forms. Eur. J. Immunol..

[25] Zeilhofer H.U., Schorr W. (2000). Role of interleukin-8 in neutrophil signaling. Curr. Opin. Hematol..

[26] Gabrilovich D.I. (2017). Myeloid-Derived Suppressor Cells. Cancer Immunol. Res..

[27] Hatzi K., Nance J.P., Kroenke M.A., Bothwell M., Haddad E.K., Melnick A., Crotty S. (2015). BCL6 orchestrates Tfh cell differentiation via multiple distinct mechanisms. J. Exp. Med..

[28] Nurieva R.I., Chung Y., Martinez G.J., Yang X.O., Tanaka S., Matskevitch T.D., Wang Y.-H., Dong C. (2009). Bcl6 mediates the development of T follicular helper cells. Science.

[29] Bentebibel S.-E., Schmitt N., Banchereau J., Ueno H. (2011). Human tonsil B-cell lymphoma 6 (BCL6)-expressing CD4+ T-cell subset specialized for B-cell help outside germinal centers. Proc. Natl. Acad. Sci. USA.

[30] Mansouri S., Katikaneni D.S., Gogoi H., Jin L. (2021). Monocyte-Derived Dendritic Cells (moDCs) Differentiate into Bcl6+ Mature moDCs to Promote Cyclic di-GMP Vaccine Adjuvant-Induced Memory TH Cells in the Lung. J. Immunol..

[31] Bunting K.L., Melnick A.M. (2013). New effector functions and regulatory mechanisms of BCL6 in normal and malignant lymphocytes. Curr. Opin. Immunol..

[32] Arima M., Fukuda T., Tokuhisa T. (2008). Role of the Transcriptional Repressor BCL6 in Allergic Response and Inflammation. World Allergy Organ. J..

[33] Hayden M.S., West A.P., Ghosh S. (2006). NF-kappaB and the immune response. Oncogene.

[34] De Goeje P.L., Bezemer K., Heuvers M.E., Dingemans A.-M.C., Groen H.J., Smit E.F., Hoogsteden H.C., Hendriks R.W., Aerts J.G., Hegmans J.P. (2015). Immunoglobulin-like transcript 3 is expressed by myeloid-derived suppressor cells and correlates with survival in patients with non-small cell lung cancer. Oncoimmunology.

[35] Liu J., Wu Q., Shi J., Guo W., Jiang X., Zhou B., Ren C. (2020). LILRB4, from the immune system to the disease target. Am. J. Transl. Res..

[36] Li S., Rouphael N., Duraisingham S., Romero-Steiner S., Presnell S., Davis C., Schmidt D.S., Johnson S.E., Milton A., Rajam G. (2014). Molecular signatures of antibody responses derived from a systems biology study of five human vaccines. Nat. Immunol..

[37] Hartnell F., Brown A., Capone S., Kopycinski J., Bliss C., Makvandi-Nejad S., Swadling L., Ghaffari E., Cicconi P., Del Sorbo M. (2018). A Novel Vaccine Strategy Employing Serologically Different Chimpanzee Adenoviral Vectors for the Prevention of HIV-1 and HCV Coinfection. Front. Immunol..

[38] Marco-Sola S., Sammeth M., Guigó R., Ribeca P. (2012). The GEM mapper: Fast, accurate and versatile alignment by filtration. Nat. Methods.

[39] Lappalainen T., Sammeth M., Friedländer M.R., Ac’t Hoen P., Monlong J., Rivas M.A., Gonzàlez-Porta M., Kurbatova N., Griebel T., Ferreira P.G. (2013). Transcriptome and genome sequencing uncovers functional variation in humans. Nature.

[40] Robinson M.D., McCarthy D.J., Smyth G.K. (2010). edgeR: A Bioconductor package for differential expression analysis of digital gene expression data. Bioinformatics.

[41] Yu G., Wang L.-G., Han Y., He Q.-Y. (2012). clusterProfiler: An R package for comparing biological themes among gene clusters. OMICS.

