# Supplementary material for: Transcriptomic Profiling of Dromedary Camels Immunised with a MERS Vaccine Candidate
Source: Vet Sci. 2021 Aug 3;8(8):156. doi: 10.3390/vetsci8080156 (PMC8402689; doi:10.3390/vetsci8080156)
Supplement: Supplementary file 1 [file vetsci-08-00156-s001.zip › Supplementary file 1.pdf]

Supplementary file

Table S1. Landscape of camel transcriptional regulation 6, 12, and 24h after injection with ChAdOx2-MERS vaccine (All comparisons are done with respect to time 0). A gene is defined as differentially regulated in a statistically significant way when abs (log_2(fold change)) ≥1 and FDR ≤0.05. All the genes in the table have abs (log_2(fold change)) ≥1; their FDR is typeset in bold whenever FDR ≤0.05, i.e. whenever differential regulation for that gene at the time point considered is statistically significant. LFC: limit fold change; FDR: false discovery rate; T=6h: study point 6 hours post vaccination; T=12h: study point 12 hours post vaccination; T=24h: study point 24 hours post vaccination.

Gene name (CamDro3 names)	Gene name (mapped to ENSEMBL human)	Gene name (InterPro)	Gene name (human Readable)	Gene description	T=6h		T=12h		T=24h	
					LFC	FDR	LFC	FDR	LFC	FDR
**Cadr_000009117**	ENSG00000168209	Q9NX09	DDIT4	DNA damage-inducible transcript 4 protein	-2.57	**0.0021**	-2.84	**0.00078**	-2.94	**0.00056**
**Cadr_000018634**	ENSG00000179094	O15534	PER1	Period circadian protein homolog 1	-1.58	**0.013**	-1.76	**0.015**	-1.96	**0.005**
**Cadr_000022112**	ENSG00000204539	Q15517	CDSN	Corneodesmosin	7.49	**0.0052**	5.91	**0.035**	7.2	**0.0053**
**Cadr_000028245**	ENSG00000182541	P53671	LIMK2	LIM domain kinase 2	2.47	**0.0072**	1.31	0.063	0.228	0.99
**Cadr_000005463**					2.23	**0.013**	1.87	0.054	0.237	0.99
**Cadr_000011526**	ENSG00000089351	Q96CP6	GRAMD1A	Protein Aster-A	1.54	**0.013**	0.845	0.072	-0.0587	1
**Cadr_000028656**	ENSG00000128789	Q969U7	PSMG2	Proteasome assembly chaperone 2	1.06	**0.013**	0.689	0.059	0.0823	0.99
**Cadr_000001885**	ENSG00000169429	P10145	CXCL8	Interleukin-8	-1.8	**0.023**	-2.01	**0.028**	-2.08	**0.015**
**Cadr_000011353**					1.52	**0.017**	0.455	0.42	-0.181	0.98
**Cadr_000002514**	ENSG00000198883	Q96PV4	PNMA5	Paraneoplastic antigen-like protein 5	3.89	**0.023**	0.915	0.61	-0.0643	1

**Cadr_000003390**	ENSG00000102312	Q9H237	PORCN	Protein-serine O-palmitoleoyltransferase porcupine	2.24	**0.023**	0.358	0.79	-0.36	0.98
**Cadr_000004852**	ENSG00000133710	Q9NQ38	SPINK5	Serine protease inhibitor Kazal-type 5	5.88	**0.023**	5.44	0.054	5.76	0.061
**Cadr_000010146**	ENSG00000135587	O60906	SMPD2	Sphingomyelin phosphodiesterase 2	1.33	**0.023**	0.583	0.22	0.227	0.97
**Cadr_000011846**	ENSG00000069399	P20749	BCL3	B-cell lymphoma 3 protein	1.19	**0.023**	0.688	0.096	0.0744	1
**Cadr_000021957**	ENSG00000096060	Q13451	FKBP5	Peptidyl-prolyl cis-trans isomerase FKBP5	-2.45	**0.023**	-2.16	0.054	-2.25	0.078
**Cadr_000023115**	ENSG00000090104	Q08116	RGS1	Regulator of G-protein signaling 1	-2.85	**0.023**	-0.514	0.73	-0.943	0.68
**Cadr_000023477**	ENSG00000133055	Q13203	MYBPH	Myosin-binding protein H	2.07	**0.023**	1.59	0.056	0.541	0.85
**Cadr_000023835**					1.47	**0.023**	0.875	0.097	0.208	0.98
**Cadr_000028639**	ENSG00000156052	P50148	GNAQ	Guanine nucleotide-binding protein G subunit alpha	1.08	**0.023**	0.656	0.094	0.261	0.91
**Cadr_000026275**	ENSG00000179361	Q8IVW6	ARID3B	AT-rich interactive domain-containing protein 3B	1.13	**0.027**	0.315	0.56	0.166	0.98
**Cadr_000007726**				hypothetical protein	1.47	**0.028**	0.717	0.2	0.72	0.43
**Cadr_000012219**	ENSG00000186818	Q8NHJ6	LILRB4	Leukocyte immunoglobulin-like receptor subfamily B member 4	1.61	**0.028**	0.934	0.12	0.186	0.99
**Cadr_000015194**	ENSG00000110195	P15328	FOLR1	Folate receptor alpha	3.16	**0.028**	1.4	0.22	0.448	0.98
**Cadr_000016822**	ENSG00000167767	Q6KB66	KRT80	Keratin, type II cytoskeletal 80	3.9	**0.034**	2.63	0.086	3.84	0.078

**Cadr_000030773**	ENSG00000008516	Q9NPA2	MMP25	Matrix metalloproteinase-25	1.87	**0.034**	0.99	0.17	-0.00766	1
**Cadr_000019826**	ENSG00000092929	Q70J99	UNC13D	Protein unc-13 homolog D	1.22	**0.036**	0.616	0.2	-0.0201	1
**Cadr_000000564**	ENSG00000113916	P41182	BCL6	B-cell lymphoma 6 protein	1.98	**0.041**	1.34	0.092	0.269	0.99
**Cadr_000001890**	ENSG00000163739	P09341	CXCL1	Growth-regulated alpha protein	-3.21	**0.07**	-5.61	**0.041**	-5.17	**0.05**
**Cadr_000002580**				hypothetical protein	1.65	**0.041**	0.922	0.18	-0.104	1
**Cadr_000004067**	ENSG00000168685	P16871	IL7R	Interleukin-7 receptor subunit alpha	-1.51	**0.041**	-1.16	0.073	-1.2	0.19
**Cadr_000010587**	ENSG00000146477	O75751	SLC22A3	Solute carrier family 22 member 3	2.2	**0.041**	0.538	0.68	0.182	1
**Cadr_000012111**	ENSG00000142512	Q96LC7	SIGLEC10	Sialic acid-binding Ig-like lectin 10	1.29	**0.041**	1.08	0.063	0.404	0.84
**Cadr_000012112**	ENSG00000129450	Q9Y336	SIGLEC9	Sialic acid-binding Ig-like lectin 9	1.76	**0.041**	1.33	0.073	0.564	0.81
**Cadr_000016314**	ENSG00000175287	Q5SRE7	PHYHD1	Phytanoyl-CoA dioxygenase domain-containing protein 1	1.88	**0.041**	1.06	0.16	-0.921	0.49
**Cadr_000016800**	ENSG00000205420	P02538	KRT6A	Keratin, type II cytoskeletal 6A	5.61	0.058	6.51	0.054	7.61	**0.041**
**Cadr_000025545**	ENSG00000186723	Q9Y4A9	OR10H1	Olfactory receptor 10H1	2.74	**0.041**	0.248	0.91	0.416	0.98
**Cadr_000027936**	ENSG00000130783	Q6P9F0	CCDC62	Coiled-coil domain-containing protein 62	1.31	**0.041**	0.899	0.096	0.734	0.41
**Cadr_000028624**	ENSG00000168454	Q86VQ3	TXNDC2	Thioredoxin domain-containing protein 2	2.18	**0.041**	1.9	0.057	1.29	0.36
**Cadr_000029654**	ENSG00000147454	Q9NYZ2	SLC25A37	Mitoferrin-1	1.87	**0.041**	1.8	0.054	0.816	0.58

**Cadr_000012282**	ENSG00000167685	Q8N0Y2	ZNF444	Zinc finger protein 444	1.71	**0.042**	0.93	0.19	0.0416	1
**Cadr_000013472**	ENSG00000175793	P31947	SFN	14-3-3 protein sigma	1.36	**0.043**	0.573	0.37	0.251	0.98
**Cadr_000015197**	ENSG00000165458	O15357	INPPL1	Phosphatidylinositol 3,4,5-trisphosphate 5-phosphatase 2	1.01	**0.043**	0.477	0.29	0.233	0.96
**Cadr_000009001**	ENSG00000276430	B3EWG5	FAM25C	Protein FAM25C	4.83	**0.047**	4.08	0.065	4.95	0.096
**Cadr_000016797**	ENSG00000205420	P02538	KRT6A	Keratin, type II cytoskeletal 6A	5.96	0.053	6.65	0.054	7.47	**0.047**
**Cadr_000025199**	ENSG00000214456	Q00G26	PLIN5	Perilipin-5	2.84	**0.048**	0.845	0.6	0.586	0.97
**Cadr_000025074**					1.12	**0.05**	0.453	0.42	0.0936	1
